# Model construction and drug therapy of primary ovarian insufficiency by ultrasound-guided injection

**DOI:** 10.1186/s13287-024-03646-y

**Published:** 2024-02-20

**Authors:** Fangfang Dai, Hua Liu, Juan He, Jinglin Wu, Chaoyan Yuan, Ruiqi Wang, Mengqin Yuan, Dongyong Yang, Zhimin Deng, Linlin Wang, Yanqing Wang, Xiao Yang, Huiling Wang, Wei Hu, Yanxiang Cheng

**Affiliations:** 1 Department of Obstetrics and Gynecology, Renmin Hospital of Wuhan University, Wuhan, 430060 Hubei China; 2 Department of Obstetrics and Gynecology Ultrasound, Renmin Hospital of Wuhan University, Wuhan, 430060 China; 3 Department of Obstetrics and Gynecology, Peking University People’s Hospital, No. 11, Xizhimen South Street, Xicheng District, Beijing, 100044 China; 4 Department of Psychiatry, Renmin Hospital of Wuhan University, Wuhan, China; 5 Department of Gynecology, Minda Hospital of Hubei Minzu University, Enshi, China

**Keywords:** Primary ovarian insufficiency, Ultrasound-guided injection, POI animal models, hUC-MSCs, Exosomes

## Abstract

**Background:**

Clinically, hormone replacement therapy (HRT) is the main treatment for primary ovarian insufficiency (POI). However, HRT may increase the risk of both breast cancer and cardiovascular disease. Exosomes derived from human umbilical cord mesenchymal stem cell (hUC-MSC) have been gradually applied to the therapy of a variety of diseases through inflammation inhibition, immune regulation, and tissue repair functions. However, the application and study of hUC-MSC exosomes in POI remain limited.

**Methods:**

Here, we first constructed four rat animal models: the POI-C model (the “cyclophosphamide-induced” POI model via intraperitoneal injection), the POI-B model (the “busulfan-induced” POI model), the POI-U model (the “cyclophosphamide-induced” POI model under ultrasonic guidance), and MS model (the “maternal separation model”). Second, we compared the body weight, ovarian index, status, Rat Grimace Scale, complications, and mortality rate of different POI rat models. Finally, a transabdominal ultrasound-guided injection of hUC-MSC exosomes was performed, and its therapeuticy effects on the POI animal models were evaluated, including changes in hormone levels, oestrous cycles, ovarian apoptosis levels, and fertility. In addition, we performed RNA-seq to explore the possible mechanism of hUC-MSC exosomes function.

**Results:**

Compared with the POI-C, POI-B, and MS animal models, the POI-U model showed less fluctuation in weight, a lower ovarian index, fewer complications, a lower mortality rate, and a higher model success rate. Second, we successfully identified hUC-MSCs and their exosomes, and performed ultrasound-guided intraovarian hUC-MSCs exosomes injection. Finally, we confirmed that the ultrasound-guided exosome injection (termed POI-e) effectively improved ovarian hormone levels, the oestrous cycle, ovarian function, and fertility. Mechanically, hUC-MSCs may play a therapeutic role by regulating ovarian immune and metabolic functions.

**Conclusions:**

In our study, we innovatively constructed an ultrasound-guided ovarian drug injection method to construct POI-U animal models and hUC-MSC exosomes injection. And we confirmed the therapeutic efficacy of hUC-MSC exosomes on the POI-U animal models. Our study will offer a better choice for new animal models of POI in the future and provides certain guidance for the hUC-MSCs exosome therapy in POI patients.

**Graphical abstract:**

The schema of construction of different animal models, extraction and identifying hUC-MSCs and exosomes, therapy of ultrasound-guided hUC-MSCs exosome injection. Note: POI: premature ovarian insufficiency; hUC-MSCs: Human umbilical cord mesenchymal stem cells; POI-C: POI-cyclophosphamide; POI-B: POI-cyclophosphamide + Busulfan; POI-U: POI-Ultrasonic guidance cyclophosphamide injection; MS: POI-Maternal separation. POI-e: ultrasound-guided hUC-MSCs exosome injection; AMH: Anti-müllerian hormone; LH: Luteinizing hormone; FSH: Follicle-stimulating hormone; DA: dopamine; T: Testosterone; PRL: prolactin; GnRH: Gonadotropin-releasing hormone.

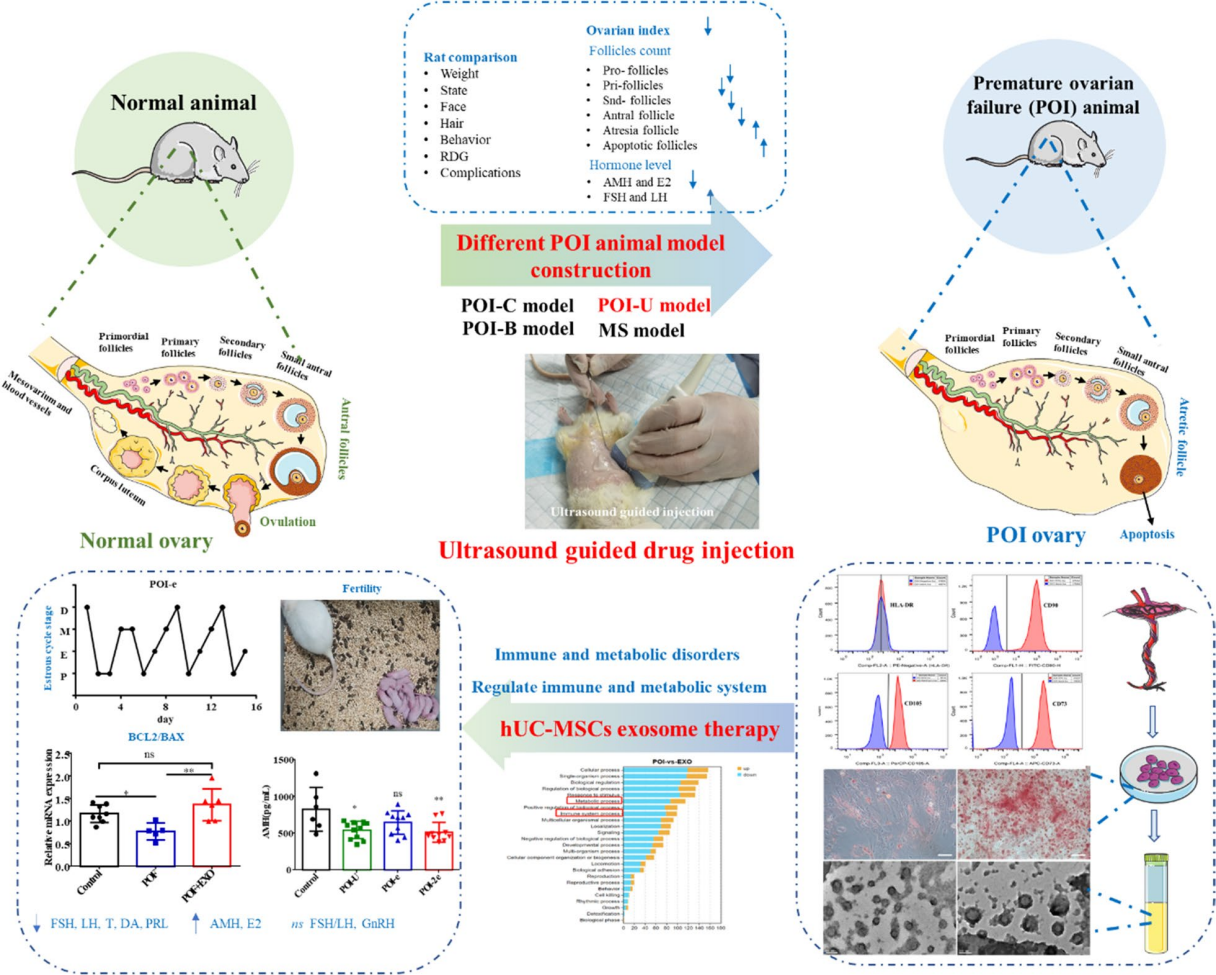

**Supplementary Information:**

The online version contains supplementary material available at 10.1186/s13287-024-03646-y.

## Introduction

Primary ovarian insufficiency (POI) affects 1–5% of women under 40 years of age [1, 2]. POI, which mainly manifests as irregular menstrual cycles, is often confirmed according to the elevated serum levels of follicle-stimulating hormone (FSH) and decreased levels of oestradiol and anti-Mullerian hormone (AMH) [3]. POI often leads to infertility, and its early onset can have a negative impact on sexual health. Additionally, long-term oestrogen deprivation has serious effects on women's health, particularly on bone density, cardiovascular and nervous systems, and sexual health [1]. Currently, clinical care for women with POI mainly includes hormone replacement therapy (HRT) and psychological support. However, an effective therapeutic solution for POI remains elusive.

POI is associated with chromosomal or genetic changes, infections, metabolic disorders, autoimmune diseases, and iatrogenic factors (ovarian surgery, radiotherapy, and chemotherapy, etc.) [1, 4]. According to the aetiology of the disease, POI animal models can be divided into the following four types: the “chemotherapy-induced” model, “autoimmune” model, “mental stress” model, and “natural ageing” model. Among these, the “chemotherapy-induced” model is the most used animal model in research. However, there are big differences between different models. Suitable and optimal animal models are essential carriers for drug development and mechanistic research. In a previous review, we compared the advantages and disadvantages of different POI animal models [5]. In this investigation, we constructed different POI animal models and evaluated their strengths and weaknesses, which included body weight and ovarian changes in rats, hormone level fluctuations, model success rate, complications, and so on. Our study aimed to build a suitable and ideal POI animal models for drug development and mechanistic research.

In recent years, human umbilical cord mesenchymal stem cells (hUC-MSCs) have attracted much attention as the potential cell therapy tools due to their proliferation, multipotency, homing/migration abilities, trophic effects, and immunosuppressive properties. These cells have been demonstrated to possess therapeutic potential in various autoimmune, inflammatory, and degenerative diseases [6]. Accumulating studies have shown that intra-ovarian transplantation of hUC-MSCs can restore fertility, recover serum hormone levels, and facilitate follicle formation in a “chemotherapy-induced” POI rat model [4, 7]. However, poor engraftment efficiency and insufficient viability of hUC-MSCs limits their application. Hence, various strategies have been developed to improve the effectiveness of hUC-MSCs. A 2022 study showed that the hUC-MSCs with autocross-linked Hyaluronic acid gel can rescue ovarian reserve and fecundity in POI and naturally aging mice [8]. In addition to physical crosslinking methods, hUC-MSCs derivatives have been extracted to restore the function of ovary of POI.

Many studies reported that factors, including cytokines and exosomes, can be secreted, which lead to the ovarian recovery of POI through reducing apoptosis and inflammation, and inducing angiogenesis [8, 9]. A recent study from 2023 has shown that small membrane-coated vesicles, namely MSCs cell exosomes, can protect granulosa cells from chemotherapy-induced damage via venous injection [10]. However, an increasing number of studies suggest that the biological distribution of hUC-MSCs in target organs via veins is rare [6]. Ultrasound-guided drug injection is an efficient and safe method [11]. This study mainly focused on the therapeutic effect of ultrasound-guided hUC-MSC exosomes on POI and explored the possible mechanism by which hUC-MSC exosomes rescue the manifestations of POI.

In this work, we found a novel POI animal model, the “cyclophosphamide-induced” POI model under ultrasonic guidance (POI-U model), which has less complications and high model success rate. Further, we successfully confirmed the therapeutic effects of hUC-MSC exosomes administered via ultrasound-guided injection in the POI-U model. Mechanistically, hUC-MSC exosomes may ameliorate ovarian function by regulating the immune and metabolic systems.

## Methods

### Animals and experimental design

**Fig. 1:**
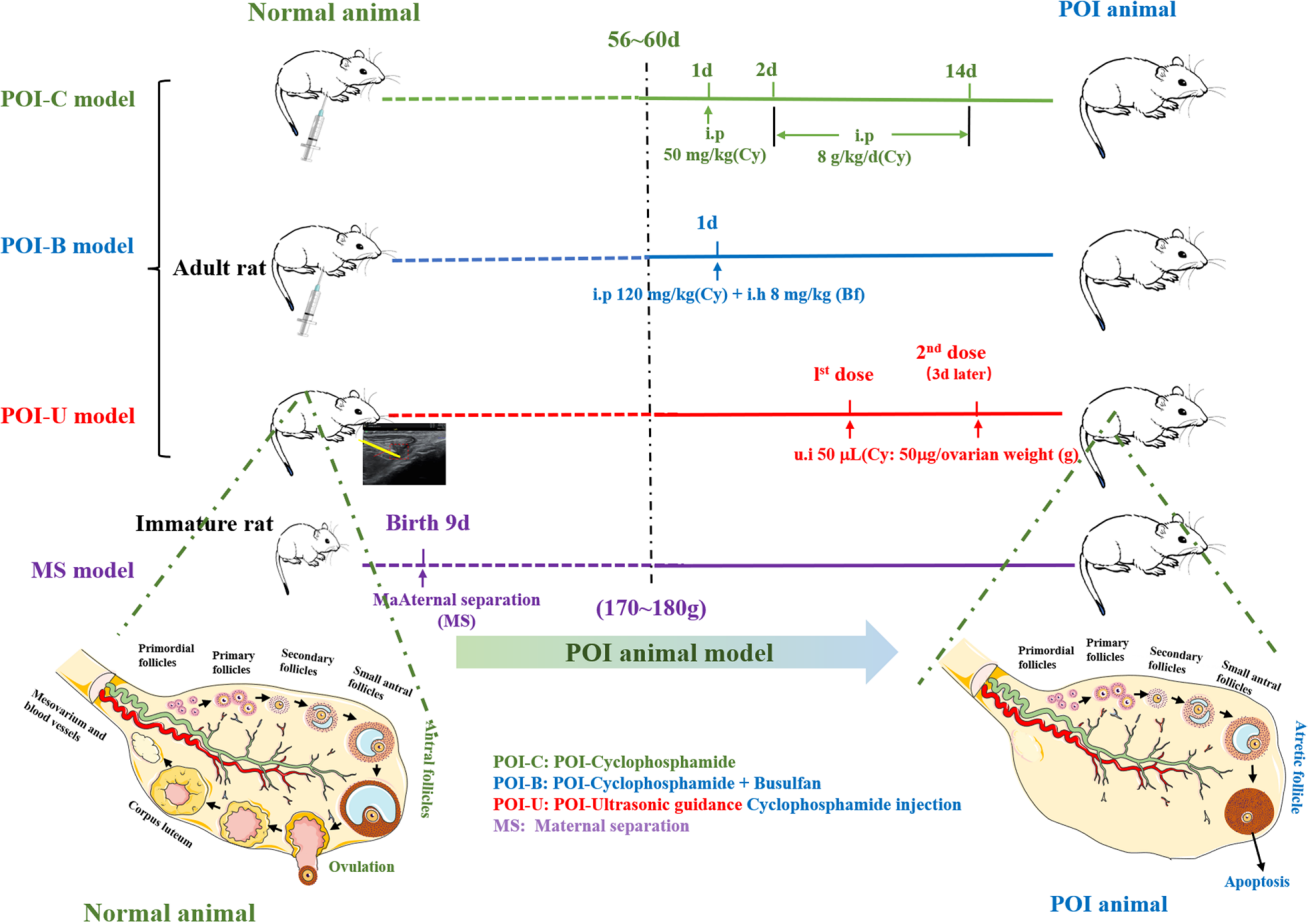
Schema of different premature ovarian insufficiency (POI) models. Note: POI-C: POI-cyclophosphamide; POI-B: POI-cyclophosphamide + Busulfan; POI-U: POI-Ultrasonic guidance cyclophosphamide injection; MS: Maternal separation

Ninety-four female Wistar rats (5 ~ 7 weeks old) were purchased from Beijing Vital River Laboratory Animal Technology Co. Ltd., China. The animals were raised in a specific pathogen-free (SPF) environment. The rats were grouped into five groups: the control group (comprising fifteen rats), the POI-C group (consisting of twenty-four rats), the POI-B group (cyclophosphamide + busulfan, containing ten rats), the POI-U group (including thirty rats), andthe MS group (also including fifteen rats), according to different model methods. In addition, there were fifteen rats in the ultrasound-guided exosome injection group (POI-e and POI-2e). The specific experimental flow chart is shown in Fig. 1.

The preparation methods of four animal disease models according to the causes of POI were selected to evaluate the feasibility of the animal models [5, 12]. The grouping was as follows: (1) POI-C model: Female Wistar adult rats aged 60 d were intraperitoneally injected with CTX (50 mg/kg) on the first day, followed by 2 weeks of CTX (8 g/kg/d); (2) POI-B model: Female Wistar adult rats approximately 60 days old were given a single dose of CTX (120 mg/kg) via intraperitoneal injection + busulfan (8 mg/kg) via subcutaneous injection; (3) POI-U model: Female Wistar adult rats approximately 60 days old were injected with CTX (50 μg/ovarin weight (g)  ~50 μL) according to the weight of ovary in both ovaries under the ultrasound guidance, and the same dose of CTX was injected under ultrasound guidance 2 weeks later; (4) MS model: On the 9 th day after birth, mother and infant rats were separated for 1 d, which simulated early life stress stimulation [13]. Relevant indicators were monitored when the weight of the young rats reached approximately 60 d. All animal studies were approved by the ethics committee for laboratory animal welfare (IACUC) of Renmin Hospital of Wuhan University [No. WDRM animal (f) No. 20210611A].

### Ultrasound guided abdominal drug injection

The adult rats were fasted for 5–6 h. The rats were anesthetized with isoflurane. Firstly, After the induction concentration was appropriately adjusted (the induction concentration of isoflurane was set to 3–4%), the induction box was filled with anesthetic for about 1 min. Then, the animal was put into the induction box, and the induction box was closed, waiting for the animal to be completely anesthetized. Secondly, after the maintenance concentration was properly adjusted (2–2.5% isoflurane for rats), the animal was removed from the induction box, and its head/nose was fixed in the anesthesia mask. Finally, after the animal experiment was completed, the evaporator was closed, and the rats were kept in pure oxygen for about 5–10 min to facilitate the rapid recovery of the animal.

After anaesthesia, the hair on the abdomen was removed using a shaving knife, a coupling agent was applied, and a low-frequency ultrasonic probe (Ultrasonic probe 11L) was applied to search for the ovaries via an ultrasound instrument (*Voluson E10*). The ovaries of normal rats appeared as low-echo liquid dark areas of 0.5–1 cm, situated adjacent to the kidneys, with abundant blood flow. Then, a 0.7 × 80 mm needle/22 g (7/22 G) was used to penetrate into the ovarian substance and both the left and right ovaries were treated with 50 μL cyclophosphamide chemotherapy drugs (50 μg/ovarian weight (g)) with fluorescent dyes and hUC-MSCs. The specific steps were as follows: (1) The operator's puncture needle was placed directly under the probe in the direction of the ovarian parenchyma to observe whether the blood flow was near the puncture needle head; (2) the operator fixed the needle position, and the assistant slowly injected 50 μL of the fluorescent chemotherapeutic drug with a 1 mL syringe; (3) the chemotherapy drug syringes was removed and the other needle with 50 μL of saline irrigate the syringes ; (4) the ovary slightly enlarged immediately after the injection, and a comparison of the ovarian diameter before and after the injection was performed; and (5) one to two animal samples were collected within 12 h post-injection, and the fluorescence intensity of the ovary was observed by an in vivo imaging apparatus. The ovarian diameter of the rats was measured under direct ultrasound. The longest and shortest diameters measured by the ultrasonic display were a cm and b cm, respectively. The area of the ovary was calculated as $\Pi *\frac{a}{2}*b/2$ cm^2^_._

### Culture and identification of hUC-MSCs

hUC-MSCs were provided from Hubei Levobank Biotechnology Co., Ltd., and Seidet Biotechnology Development Co., Ltd. The cells were cultivated using a specialized serum-free stem cell culture medium supplemented with a primary cell culture additive (cat: NC0103, Youkang Biotechnology (Beijing) Co., Ltd.). The morphology of hUC-MSCs was photographed during the first 1–3 generations. Lipogenesis was induced in hUC-MSCs as follows: (1) when hUC-MSCs reached 80 ~ 90% confluence, digestion was performed. (2) hUC-MSCs were inoculated into six-well plates at approximately 5 × 10^4^ cells/well and 2 mL/well hUC-MSC complete culture medium. (3) The fluid was changed every three days (with complete culture medium of stem cell) until the cells reached 100% confluence. (4) Once the cells reached 100% confluence, the old culture medium was removed, and lipogenic inducer A (2 mL/well) was added for induction. (5) Three days later, lipogenic inducer B was exchanged for maintenance. Twenty-four hours later, lipogenic inducer A was exchanged for induction, and four-five cycles were carried out. (7) Oil red O staining was performed to stain lipid droplets. Osteogenesis was induced in hUC-MSCs. Specifically, (1) when hUC-MSCs reached 80 ~ 90% confluence, they were digested; (2) hUC-MSCs were inoculated into six-well plates at approximately 4 × 10^3^ cells/well, and 2 mL/well hUC-MSC complete culture medium was added, which was incubated at 37 °C and 5% CO_2_. (3) After 24 h, the old culture medium was removed, and pore osteogenic induction solution (2 mL) was added. (4) The pore-osteogenic induction solution was changed every three days for 2–3 weeks. (5) After 2–3 weeks, calcium nodules were formed, and alizarin red staining was performed. hUC-MSC markers (FITC-CD90, CD105-PerCP-Cy™5.5, CD73-APC, HLA-DR-PE) were detected by flow cytometry (Beckman Coulter, CA, USA).

### Extraction and characterization of hUC-MSC exosomes

After the supernatant from serum-free culture medium was collected, it was concentrated using a 100 kDa ultrafiltration tube and then centrifuged at 4 °C at 500 × g for 10 min, 2000 × g for 10 min and 20,000 × g for 20 min to remove cell debris and microvesicles. The samples were then centrifuged at 100,000 × g for 120 min to remove the supernatant. After DPBS resuspension and centrifugation at 100,000 × g for 120 min, exosomes were obtained by transparent precipitation (Cat: OptimaXE-100, Beckman Coulter) to extract the hUC-MSC exosomes. Then, 50 μL of rhodamine working liquid was added to every 100 μg of exosomes (as calculated by the BCA method), and the exosomes were evenly blown and mixed using a vortex oscillator for 1 min, incubated at room temperature for 10 min, and resuspended in 1X PBS. The exosomes were re-extracted by the overspeed centrifugal method to remove excess dye. Rhodamine-labelled exosomes were used. Note that the above operation should strictly avoid light, and rhodamine working liquid should be available. An equal amount of PBS was slowly injected into the ovarian tissue of the rats as a control group during the ultrasound-guided drug injection.

The morphology of hUC-MSC exosomes was observed by transmission electron microscopy (TEM, JEM-2100, JEOL, Japan). First, 20 μL of exosome-suspended droplets was added to the electron microscope copper mesh and left to stand for more than 1 min. Then, the sample was fixed with 2% phosphotungstic acid solution for 1 ~ 10 min and air-dried at room temperature. Finally, the sample was observed and photographed under a biological transmission electron microscope. The zeta potential and particle size of hUC-MSC exosomes were detected by a Zeta sizer Nano ZS (Malvern, UK). The zeta potential analyser was preheated for 30 min in advance, and an appropriate amount of exosome suspension was added into the quartz colorimetric plate to detect the zeta potential to test the charge of the exosomes. Each sample was assayed at least 3 times.

### Detection of body and ovarian weight of rats

The body weight of the rats was continuously monitored every day. The rats were weighed gently to minimize stress. After the rats were killed using sodium thiopental (50 mg/kg, i.p.), the volume of the drug injected into the rat was about 0.5 mL. The skin and muscle layers were cut from the abdomen to extract ovarian tissues. Their ovarian tissue was separated, and the surrounding adipose tissue was carefully stripped away for weighing. The ovary index was calculated as ovarian weight (mg)/rat weight (g) × 100% [12]. The ovarian tissue to be fixed was carefully trimmed and fixed in 4% paraformaldehyde for tissue sectioning. The tissues used for mRNA detection were directly frozen in liquid nitrogen and then transferred to − 80 °C cryogenic refrigerator.

### Examination of oestrous cycles

A daily vaginal smear was performed from 8:00 to 9:00 AM. Specifically, we gently rinsed the rat's vagina with 7 μL of sterile normal saline until the liquid became slightly cloudy and then uniformly spread onto a slide. After the slides were air-dried, they were immersed in absolute ethanol for 10 min and then dehydrated in gradient alcohol. Haematoxylin was added at 37 °C for 10 min and rinsed off with running tap water for 1 min. Subsequently, the slides were dyed with eosin staining for 30 s ~ 1 min and repeatedly rinsed with running tap water. The slides were observed under a light microscope after completely drying (Olympus BX53; Olympus Corporation).

### HE staining and counting of the ovarian follicles

Ovarian tissue was removed from the paraformaldehyde fixation solution and then transferred to dissolved paraffin for fixation after transparent dehydration. Subsequently, 4 μm thick tissue was cut from the ovarian centre to make sections. Sections were stained with haematoxylin for 5 min and then rinsed with running water several times. Hydrochloric acid alcohol was used for differentiation for 1 s, washed in warm water for a few seconds, and then cleaned with distilled water at room temperature for 1 s. Finally, the slides were soaked in eosin solution for 40 s and rinsed for 2 s, dehydrated with gradient ethanol, cleared with xylene, and finally sealed with neutral rubber.

The number of follicles in each ovary in different groups was counted. We counted ovarian follicles in the following categories: Follicles at different developmental stages were divided into primordial follicles, primary follicles, secondary follicles, and sinus follicles. Primordial follicle: A single oocyte is surrounded by a layer of flattened granulosa cells. Primary follicle: The oocytes are surrounded by a single layer of cubic granulosa cells. Secondary follicle: The oocytes are surrounded by more than two layers of granulosa cells, with no follicular cavity. Sinus follicles: The follicles are further enlarged, and follicular spaces are visible. Atretic follicle: The shape of the follicle is irregular, and the oocyte has severe nuclear deviation, shrinkage, zona pellucida collapse, loose granulosa cells and follicular membrane cells, and cells that have atrophied and shed into the follicular antrum.

### Enzyme-linked immunosorbent assay

When the rats were anaesthetized, a disposable serum separation tube was used to immediately take blood from the heart at the peak of the heartbeat (3.5–5 mL) (Additional file 1: Fig. S1). The procedure for taking blood from the heart is shown in Additional file 2: video 1. After the blood collection vessel was inverted, it was left for 1 h at room temperature. Then, the sample was centrifuged at 3000 rpm for 15 min, and the supernatant was collected and stored at − 80 °C in separate containers to avoid repeated freeze‒thaw cycles. Samples with haemolysis need to be discarded. Serum hormone levels (E2, FSH, T, LH, AMH, GnRH, PRL, DA) were detected by enzyme-linked immunosorbent assays. This procedure was repeated three times for each sample according to the corresponding kit instructions (cat: ELK1208, ELK1315, ELK1332, ELK2367, ELK4910, ELK5453, ELK7644, ELK7879, ELK Biotechnology, Wuhan, China).

### Fertility test

After modelling, female rats in different groups were mated with 3-month-old male rats in a 2:1 cage. The male rats were separated out after 14 d of cages, and the expected delivery date was 21 d. The production of rats was observed from the 18th d, and litter size was counted for comparison between groups.

### Real-time qPCR

Frozen ovarian tissue was removed and ground into powder in liquid nitrogen. TRIzol (1 mL, Shanghai Yisheng Co., Ltd.) was added to ovarian tissues. Then, RNA was extracted through a series of steps via chloroform, isopropyl alcohol, and ethanol. Finally, the RNA was dissolved in DEPC water. The concentration was determined with a NanoDrop 2000, and reverse transcription was performed according to the instructions of a reverse transcription kit. Quantitative PCR was performed according to the quantitative PCR kit instructions (Shanghai Yisheng Co., Ltd.). Table 1 lists the primers. The relative levels of genes were calculated using the 2^−△△Ct^ method.

**Table 1:** lists the primers that were utilized

Primers	Sequence	
r-gadph	F	ATGGCTACAGCAACAGGGT
	R	TTATGGGGTCTGGGATGG
r-*bax*	F	GAGGTCTTCTTCCGTGTGG
	R	GATCAGCTCGGGCACTTT
r-*bcl2*	F	AGGAACTCTTCAGGGATGG
	R	GCGATGTTGTCCACCAG

### RNA-seq analysis

In each group of rats (POI and POI-e), ovarian tissue samples were randomly selected from three rats. RNA-seq was performed by Guangzhou Jidio Biotechnology Co., Ltd. Specially, Specially, total RNA was extracted using the Trizol reagent (Invitrogen, Carlsbad, CA, USA). Total RNA (1 μg per sample) was used to construct sequencing libraries. Briefly, RNA quality was evaluated by an Agilent 2100 Bioanalyzer (Agilent Technologies, Palo Alto, CA, USA) and checked using RNase free agarose gel electrophoresis. After total RNA was extracted, the mRNA was enriched by Oligo (dT) beads. Then the enriched mRNA was fragmented into short fragments using a fragmentation buffer and reversely transcribed into cDNA by using a NEB Next Ultra RNA Library Prep Kit for Illumina (NEB #7530, New England Biolabs, Ipswich, MA, USA). The purified double-stranded cDNA was end-repaired, A-tail was added, and sequencing joints were connected. cDNA of about 200 bp was screened by AMPure XP beads (1.0X) for PCR amplification, and PCR products were purified by AMPure XP beads again. Finally, the resulting cDNA library was sequenced using Illumina Novaseq 6000 by Gene Denovo Biotechnology Co. (Guangzhou, China). Sequencing mode is double-ended sequencing 2 × 150 bp (PE 150), read length 150 bp.

The sequencing fragment process was obtained during sequencing, and each fragment is called a read. The reads were further filtered by fastp (V 0.18.0) to obtain quality clean reads. The steps for filtering reads are as follows: (1) reads containing adapter were removed; (2) reads containing more than 10% N were removed; (3) Remove reads that are all A-base; (4) low-quality reads (base numbers with mass value Q ≤ 20 account for more than 50% of the entire read) were removed.

Clean reads were then mapped to the rattus morvegicus reference genome (Ensembl_release106) using HISAT (v2.2.4). Then, the mapped reads were assembled by using StringTie (v1.3.1). The gene expression differences between POI group and POI-e group were evaluated by the fragment per kilobase of transcript per million mapped reads method (FPKM). The calculation method of FPKM is $FPKM=\frac{10^6 C}{NL/10^3}$ where C is the number of fragments (count) to be compared to the gene, N is the total number of fragments to be compared to the reference gene, and L is the effective length of the gene.

Differentially expressed genes (DEGs) were identified using DESeq2 software between two different groups. Fold change (FC) and difference significance were used to screen the DEGs. DEGs with FC value greater than 2 or lower than − 2, and a P value lower than 0.05 were considered significant. Finally, GO function annotation and KEGG pathway enrichment analysis were further carried out for DEGs via DAVID database (https://david.ncifcrf.gov/).

### Statistical analysis

The experimental results were obtained by GraphPad Software (Version 7.0, United States). The measurement data were expressed as mean ± standard deviation. The independent sample t test was used to compare the normal distribution data between the two groups. The LSD test in the one-way analysis of variance was used to compare the normal distribution homogeneity of variance data between the three groups. *P* < 0.05 indicates statistical difference.

## Results

### Comparison of body weight, ovarian index, and status of rats in four animal models

**Fig. 2:**
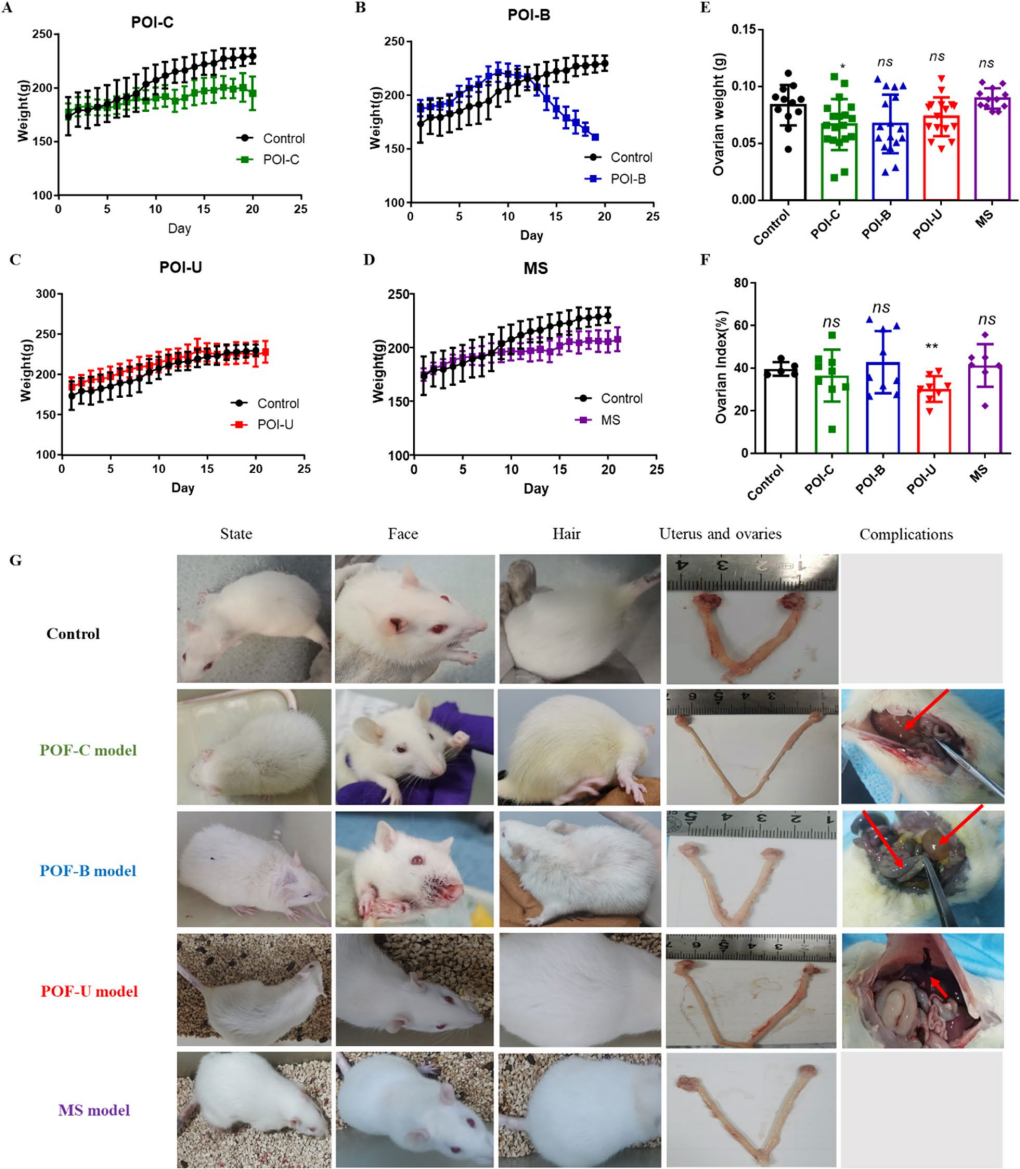
Comparison of weight, ovarian index, and status of rats in four animal models. **A**–**D** Body weight changes of POI-C, POI-B, POI-U and MS groups; **E**–**F** Ovarian weight and index of POI-C, POI-B, POI-U and MS groups; **G** Comparison of status, expression, hair, ovary, and complications of rats in Con, POI-C, POI-B, POI-U, and MS groups

The changes of body weight in the four models (POI-C, POI-B, POI-U, and MS) were continuously monitored throughout the modelling period. We found that the body weight of the rats exhibited a stable increase in the initial stage. While, with the accumulation of drug concentrations in the body, the body weight of the rats in the POI-C and POI-B groups gradually decreased, especially in the POI-B group at approximately 12th day of administration (Fig. 2A–D). The phenomenon maybe that systemic administration of chemotherapeutic drugs over a period can lead to systemic malnutrition. The above findings indicate that chemotherapy drugs had adverse reaction when they were administrated throughout the body. It may be because that the systemic administration of chemotherapy drugs will damage to stomach, digestive tract, liver, and kidney, etc., resulting in loss of appetite, emaciation, and other adverse reaction [14]. After the rats were sacrificed, we compared the ovarian weights of rats. The results displayed that the ovarian weight of rats in the POI-C group was decreased significantly, while the ovaries weight of rats in the POI-B, POI-U, and MS groups remained unchanged. However, the ovaries weight of rats in the POI-B and POI-U groups showed a declined trend (Fig. 2E). We further compared the ovarian index, and the results indicated that the POI-U group showed a highly decreased index, while other POI models had no significant changes (Fig. 2F). The higher the value of the ovarian index, the better the quality of the ootid and the better the ovarian function, which is the best period of conception. On the contrary, the poor quality of the ootid and the gradual decline of the ovarian function. The results showed that POI-U animal has a huge damage to ovary compared with other animal models, which maybe that local injections of chemotherapy drugs have more damage to the ovaries than systemic administration.

The status (RGS pain score, activity, expression, hair), complications, and mortality of rats can indirectly reflect the effect of models. We used the Rat Grimace Scale (RGS) scale to score the pain of the rats [15]. The higher the RGS score was, the worse the condition of the rats were. The results showed that the RGS score of rats was 0 in the POI-U and MS groups. However, the cumulative RGS scores of the POI-C and POI-B groups were four and three, respectively (Table 2). In addition, the rats were very active with shiny fur in the POI-U and MS groups. However, the rats were depressed with dull fur and a dishevelled appearance in the POI-C and POI-B groups (Fig. 2G). The reason maybe that systemic administration increased pain and systemic adverse reactions in rats.

**Table 2:** The behavior and complications of different POI rat models

Group	Social behavior	Appearance	RGS				Hair	Ovaries weight	Complications	Death rate (%)
			Orbital tightening	Nose/cheek flattening	Ear changes	Whisker change				
Control	Active	Normal	0	0	0	0	Smooth, lightness	0.083 ± 0.005 g	/	0
POI-C model	Dispirition	Distress	2	1	1	0	Rough, dinginess,	0.068 ± 0.026 g	Bone marrow suppression; Hepatic nodules; bleeding (Nasal, orbital, Intestine)	37.5
POI-B model	Dispirition	Distress	1	1	1	0	Rough, dinginess	0.07 ± 0.28 g	Urinary retention, bleeding (Nasal, orbital, Intestine)	40
POI-U model	Active	Normal	0	0	0	0	Smooth, lightness	0.074 ± 0.016	Injured blood vessels	9
MS model	Active	Normal	0	0	0	0	Smooth, lightness	0.089 ± 0.008 g	Mental disorder (Schizophrenia or depression)	0

NC3Rs: National Centre for the replacement Refinement & Reduction of Animal in Research; POI-C (cyclophosphamide); POI-B (Busulfan); POI-U (Ultrasound-guided cyclophosphamide injection); RGS: Rat Grimace Scale

For complications, there is a risk of bleeding at injection site in the POI-U group. Rats in the MS group had obvious early life stress. In addition, the rats in the POI-C and POI-B groups showed a tendency to haemorrhage (such as nostril, orbital and intestinal haemorrhage). Some rats in the POI-C group showed myelosuppression and hepatic nodules. Two rats in the POI-B group had urinary retention complications. Notably, the mortality rates of the POI-C, POI-B, POI-U and MS groups were 0%, 37.5%, 40% and 9%, respectively. The ovarian weights of rats in the Control, POI-C, POI-B, POI-U and MS groups were 0.080 ± 0.005 g, 0.068 ± 0.026 g, 0.07 ± 0.028 g, 0.074 ± 0.016 g and 0.089 ± 0.008 g, respectively (Table 2**)**, which may due to that Chemotherapy drugs do more damage to the ovaries than early life stress. Above funding showed that POI-U and MS animal had less complications and mortality, which is a relatively safe way to construct animal models.

### Comparison of ovarian follicles and hormone levels in the four animal models

**Fig. 3:**
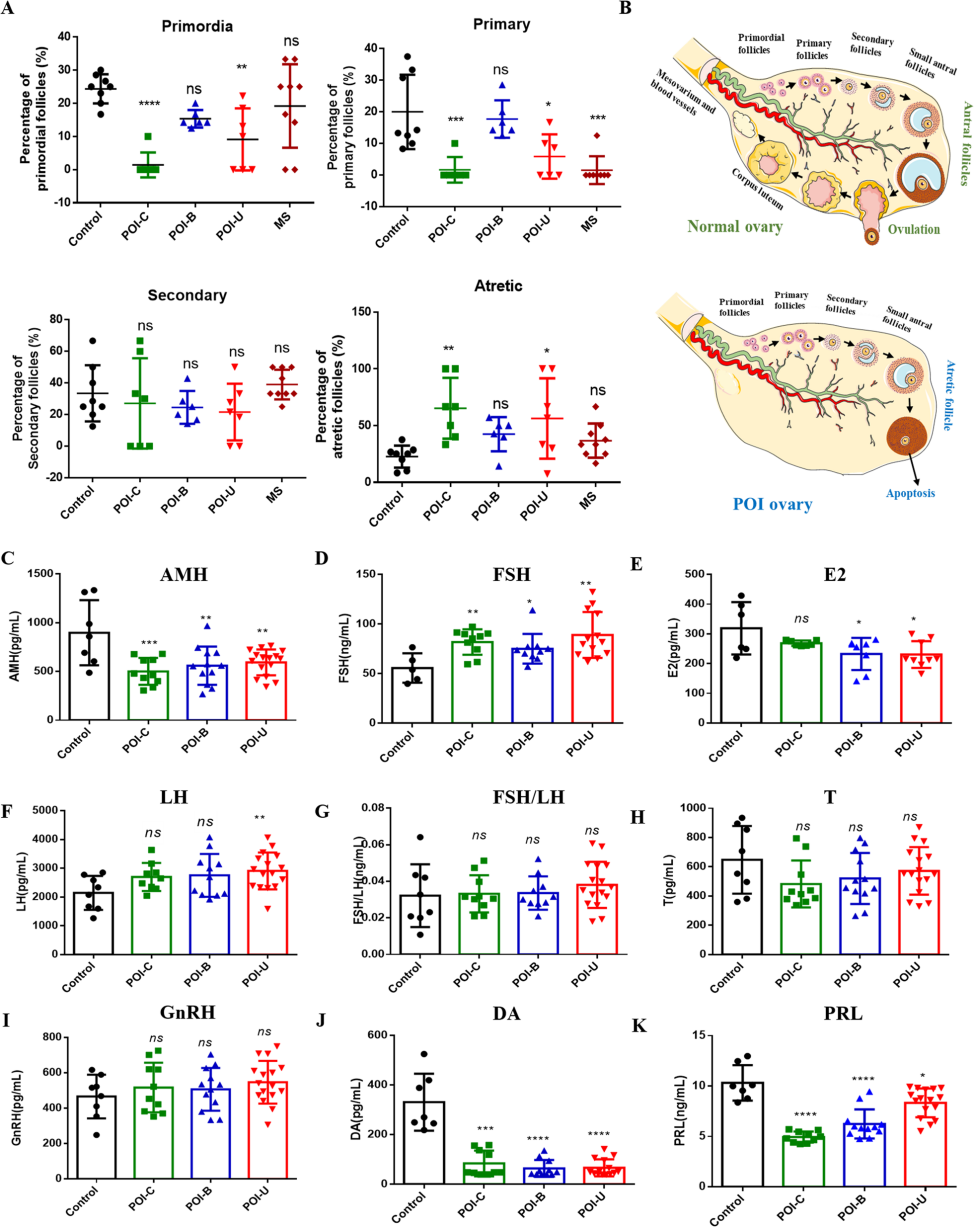
Comparison of ovarian follicles and hormones levels in four animal models. **A**, **B** Comparison of rat ovarian follicles in Con, POI -C, POI-B, POI-U and MS groups. **C**–**K** Comparison of serum AMH, FSH, LH, E2, FSH, FSH/LH, T, GnRH, DA and PRL in Con, POI -C, POI-B, POI-U and MS groups

Follicular development can be divided into four stages: primordial follicles, primary follicles, secondary follicles, and mature follicles. At primordial follicle, a single oocyte is surrounded by a layer of flattened granulosa cells. At primary follicle, the oocyte is surrounded by a single layer of cubic granulosa cells. At secondary follicle, the oocyte is surrounded by large granulosa cells arrenged in two layers, with no follicular cavity. At sinus follicles, the follicles are further enlarged, and follicular spaces are visible. Changes in follicles can also be evaluated as indicators of POI in animal models. We further compared the follicular changes in the four animal models. The results showed that the number of atretic follicles increased significantly in the POI-C and POI-U groups, while there was no significant change in the POI-B and MS groups (Fig. 3A, B). The reason maybe that CTX is more injurious to the follicles than busulfan. The results showed that cyclophosphamide have indeed huge damage to the ovarian follicular.

At present, the diagnosis of POI is mainly relies on alterations in hormone levels. We further compared the serum levels of hormones in the four animal models. Overall, compared with those of the control group, serum AMH, E2, DA and PRL levels were significantly decreased in the POI-C, POI-B and POI-U groups. Conversely, the serum levels of FSH and LH were highly increased, and the change in the POI-U group was the most obvious. There was no significant difference in FSH/LH, T or GnRH levels in the four animal models (Fig. 3C–K). The reason may be that the blood–brain barrier blocks the penetration of drugs into the brain [16]. GnRH secreted by the hypothalamus, FSH and LH produced by pituitary gland remain unaffected in the POI-C, POI-B and POI-U groups. However, compared with those of the control group, serum FSH was decreased, while GnRH, T and DA were increased in the MS group (Additional file 1: Fig. S2), which matched well with early life stress model. MS serves as the model of early life stress, which can lead to the schizophrenia-like phenotypes and persistent brain abnormalities [17]. In the study of MS model, there was a significant increasing of DA level in MS rat [13]. Our study of MS model meets well with others investigations about early life stress. In summary, the POI-C, POI-B, and POI-U animal models were successfully constructed, while the MS model was unsuccessful.

### Characterization of hUC-MSCs and exosomes

**Fig. 4:**
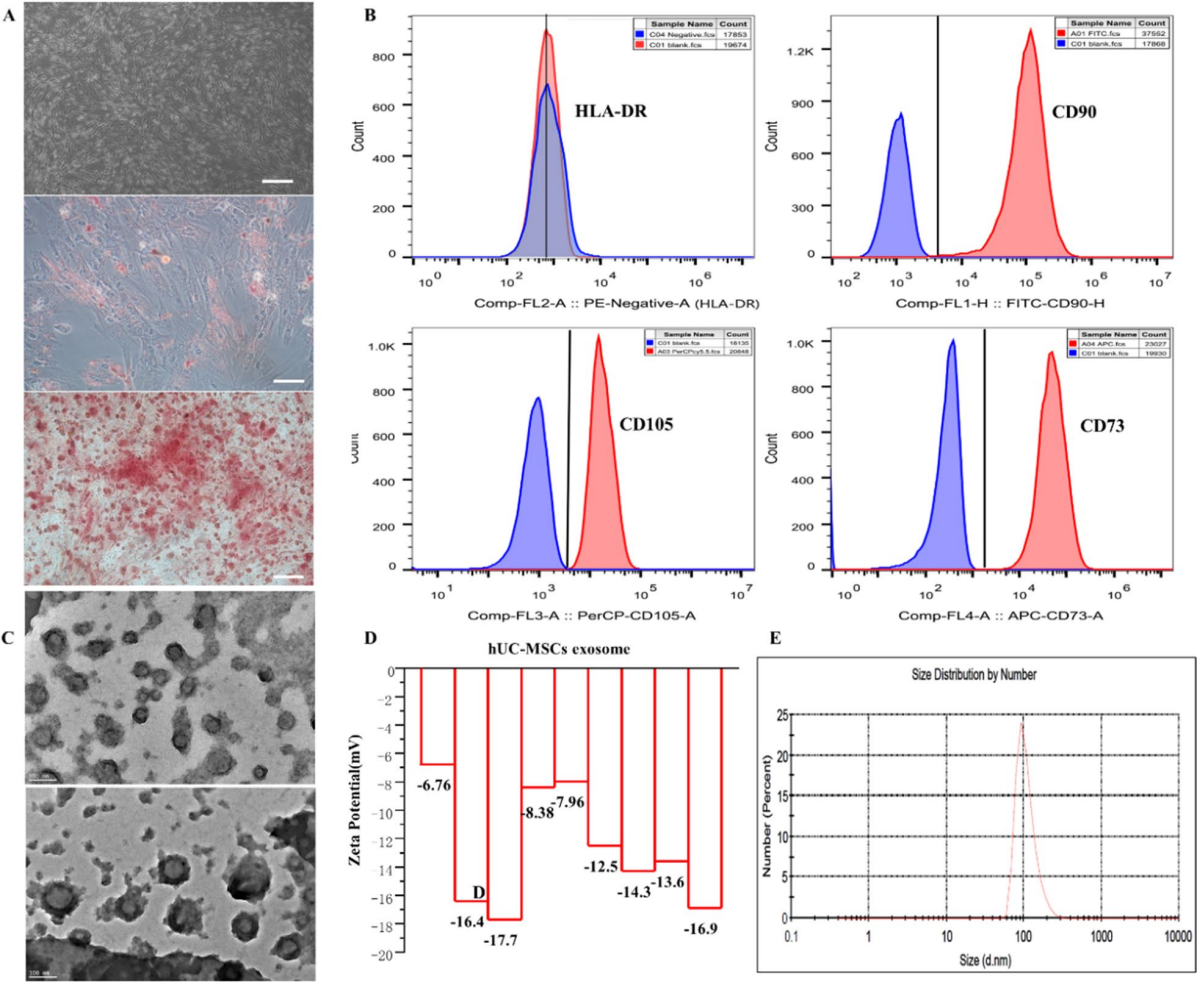
Characterization of hUC-MSCs and exosomes. **A** Morphology of hUC-MSCs in primary passage, Lipogenic and osteogenic morphology of hUC-MSCs; **B** HLA-DR, CD90, CD105, and CD73 expression of hUC-MSCs via flow cytometer; **C** The morphology of hUC-MSCs exosomes via TME; **D** Zeta detects the charge of exosomes; **E** The particle size of exosomes was detected by DLS

hUC-MSCs exhibit a fibroblast-like growth pattern, with cytoplasm protruding outwards with protrusions of different lengths. After passage, the cells demonstrate a homogeneous and swirl-like morphology and have strong adherence. hUC-MSCs successfully induced lipogenic and osteogenic differentiation in vitro (Fig. 4A). The positive rates of CD105, CD73 and CD90 in hUC-MSCs were ≥ 90%, and HLA-DR was negative (Fig. 4B). The morphology of hUC-MSC exosomes was detected by TEM. The exosomes showed a homogeneous bilayer structure. Zeta potential analysis showed that the exosomes were negatively charged. The diameter of the exosomes was approximately 100 nm (Fig. 4C–E). The results confirm that the hUC-MSCs exosomes have been successfully extracted and identified.

### hUC-MSC-exosomes were injected under ultrasound guidance

**Fig. 5:**
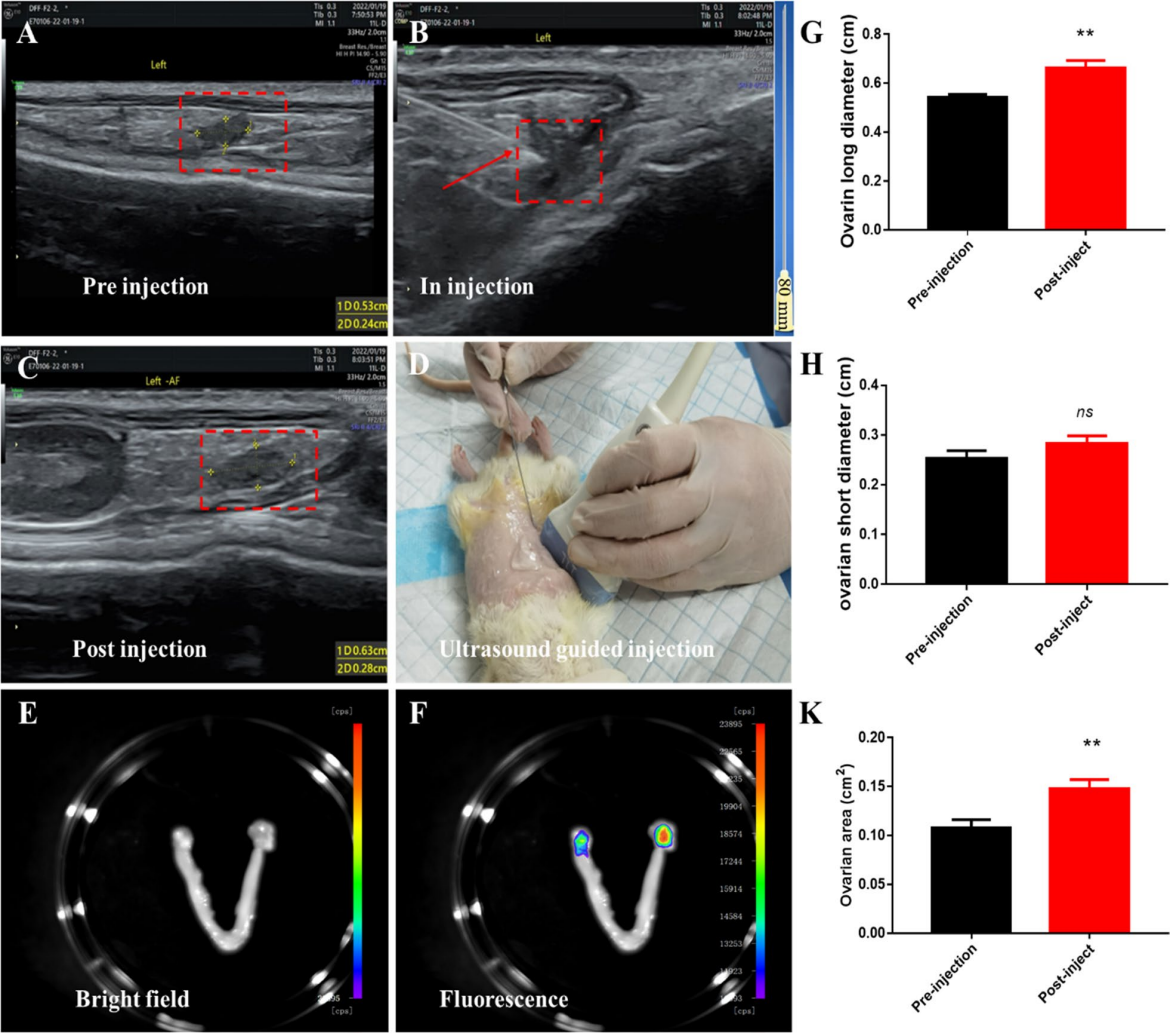
The process of hUC-MSCs-exosomes were injected under ultrasound guidance; **A**–**C** Imaging changes of ovaries before, during and after ultrasound-guided injection; **D** Image of an ultrasound guided ovarian injection; **E**, **F** Changes of ovarian fluorescence observed by in *vivo* imager

Ultrasound-guided hUC-MSC exosome injection was performed by a skilled sonographer. The ultrasound image confirmed that the needle was successfully reached to the ovarian centre of the rat. At post-injection, the ovarian diameter is directly increased compared with that pre-injection state (Fig. 5A–D). At 2 h post-injection of exosomes with fluorescent, the rats were sacrificed, and the ovarian changes of rat were observed via IVIS Spectrum CT. Distinct red fluorescence was found in the ovaries (Fig. 5E, F). By comparing ovarian diameter and area, we found that the ovarian long diameter at pre- and post-injection was 0.5433 ± 0.006667 cm and 0.6633 ± 0.01764 cm, respectively (*P* < 0.05). The short diameters at pre- and post-injection were 0.2533 ± 0.008819 cm and 0.2833 ± 0.008819 cm, respectively (*P* > 0.05). The ovarian areas at pre and post-injection were 0.1081 ± 0.004828 cm^2^ and 0.1475 ± 0.00561 cm^2^, respectively (*P* < 0.05) (Fig. 5G–K). The reason is that drug-injection slowly diffuses in the ovary, which indicated the drug was successfully injected into ovary of rat. The whole process of ovarian puncture is shown in Additional file 3: video 2**.** The ultrasound with hypoechoic shadow indicated that the drug had been slowly injected into the ovaries, which is consistent with the criteria of drug injection under ultrasound guidance [18] The above results indicated that our ultrasound-guided ovarian drug injection was wonderful.

### The effect of ultrasound-guided hUC-MSC exosome injection on rats in POI-U group

To evaluate the impact of hUC-MSC exosome injection on rats with POI, our study also compared the variations of hormone levels in rats with single-injection hUC-MSC exosomes (POI-e) and double-dose exosomes (POI-2e). The results showed that compared with those of the POI-U group, the levels of FSH, LH, T, DA, and PRL decreased, and AMH and E2 levels increased in the hUC-MSC injection groups (POI-e and POI-2e), while there was no significant difference observed in FSH/LH or GnRH. The reason maybe that hUC-MSC exosome can promote ovarian proliferation and increased synthesis and secretion of steroid hormones [19]. The fundings demonstrated that hUC-MSC exosome can effectively rescue the hormone imbalances. Moreover, there was no substantial difference in hormonal changes between the POI-e and POI-2e groups (Additional file 1: Fig. S3). That may be that single intra-ovarian administration is adequatet to achieve a therapeutic effect.

**Fig. 6:**
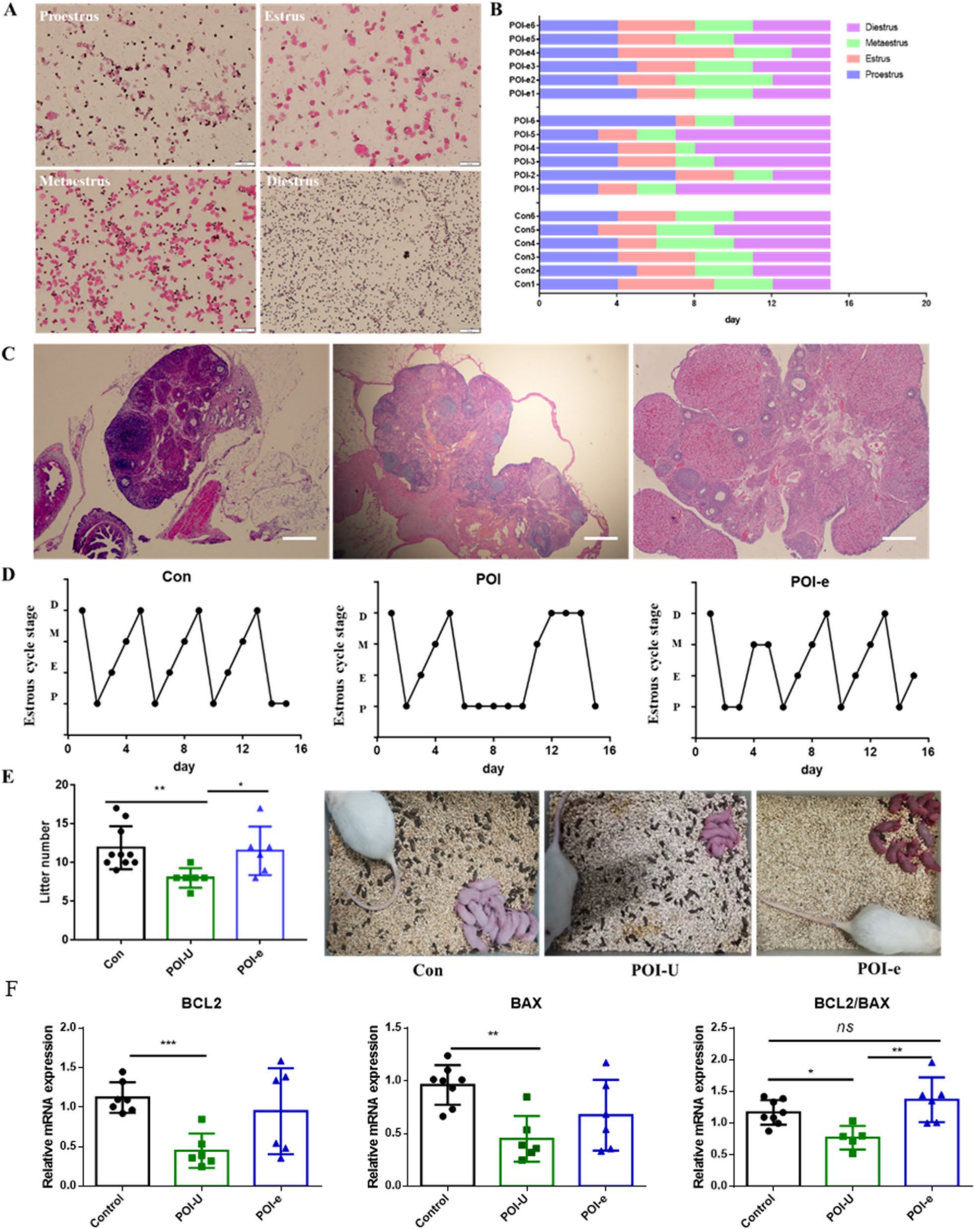
The effect of ultrasound-guided hUC-MSCs exosome injection on POI-U rats. **A** Representative photographs for proestrus, estrus, metestrus and diestrus are shown (100 ×); **B** Duration of estrous cycle stage in Con, POI, and POI-U groups. **C** HE staining of rat ovarian tissue in Con, POI, and POI-U groups; **D** Changes of estrous cycle in Con, POI, and POI-U groups. P: proestrus, E: estrous, M: metaestrus, D: Diestrus; **E** Statistics of Litter number of rats; **F** The *BCL2* and *BAX* mRNA expression in Con, POI, and POI-U groups

In proestrus, it mainly comprised nucleated epithelial cells accompanied by a few keratinocytes and leukocytes. In oestrous, a considerable number of defoliated keratinized epithelial cells and a small proportion of nucleated epithelial cells were noted. In the metestrus period, keratinized epithelial cells, nucleated epithelial cells and white blood cells were observed. In the diestrus period, many white blood cells and mucus and occasionally nuclear epithelial cells were observed. The oestrus cycle statistics showed that the oestrus cycle of the rats with POI mostly stayed in prooestrus and metaoestrus, with less dioestrus. In contrast, the POI-e-treated rats spent less time in diestrus. The number of litters was 11.9 ± 0.875, 8 ± 0.51, and 11.5 ± 1.28 in the Con, POI, and POI-U groups, respectively (Fig. 6A–E). *Eslami et al*. [20] also reported that transplantation of cMSCs restored fertility in POI mouse models. Altogether, these results suggested that POI-e treatment effectively rescued ovarian function and fertility.

To evaluate the impact of exosome injection on the level of ovarian apoptosis of POI-U rats, we assessed the ovarian apoptosis levels, as another indicator of POI in rats, in rats in the Con, POI-U and POI-e groups. BCL2/BAX was decreased in the POI-U group compared with the Con group. The higher the ratio of BCL2/BAX is, the stronger the anti-apoptosis ability is. Above findings manifested that chemotherapy drugs have a huge damage to the ovary and promote ovarian apoptosis. These results confirmed that the POI model has been successfully established. However, BCL2/BAX was significantly increased in the POI-e group **(**Fig. 6F). The results showed that hUC-MSC exosomes substantially mitigated ovarian damage in the POI-U group. Our result is consistent with other studies that exosomes can promote ovarian granulosa cell proliferation in ovaria-associated diseases [21]. To sum up, our data demonstrated that hUC-MSC exosomes can effectively restore the ovarian function and fertility of POI rat.

### The potential mechanisms underlying hUC-MSC exosome treatment in rats with POI

**Fig. 7:**
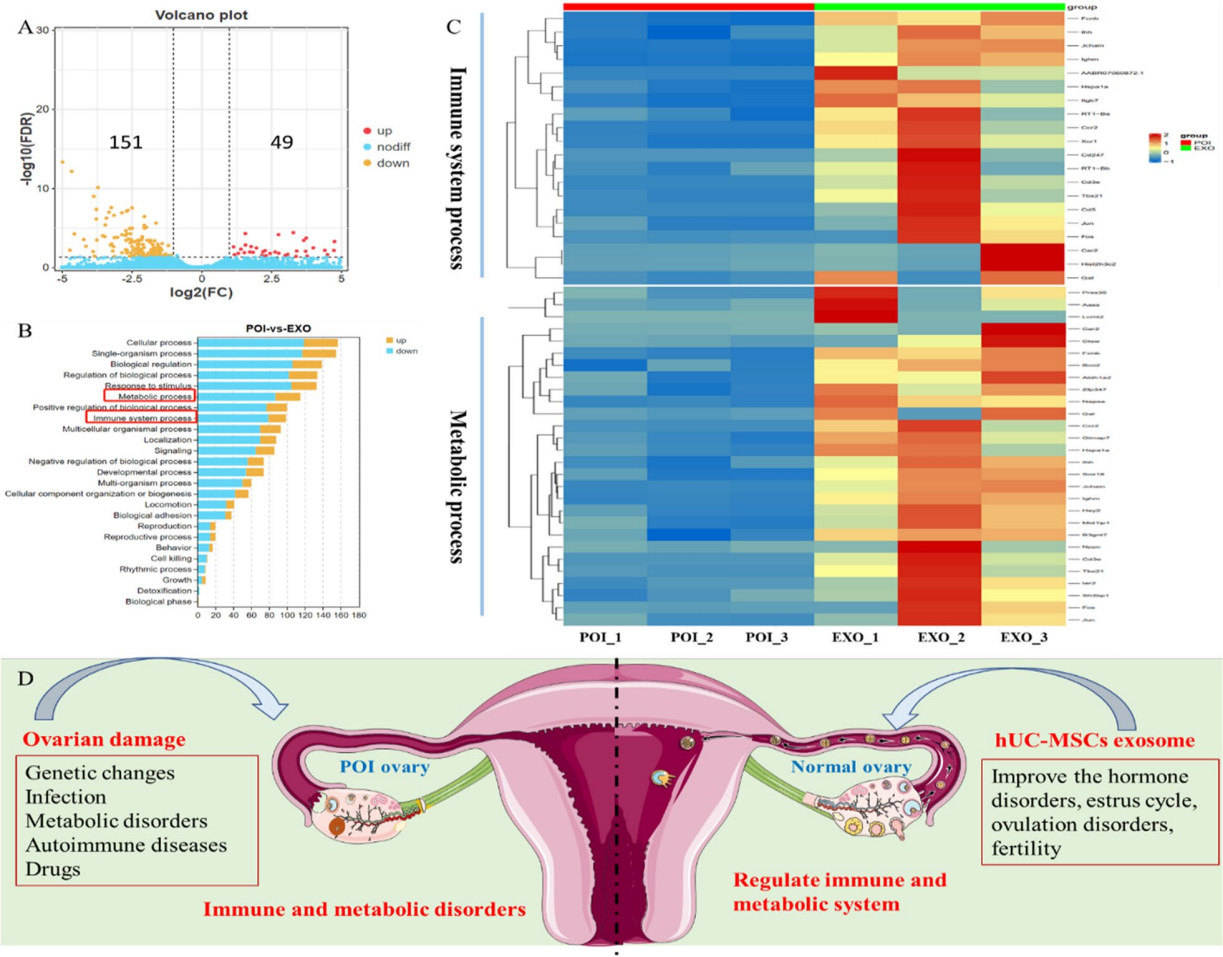
Gene analysis and mechanism of the POI-U model and hUC-MSCs exosome treatment. **A** The volcano maps of upregulated and downregulated gene; **B** GO pathway of DEGs; **C** Volcanic map of immune and metabolic pathways from DEGs; **D** Schematic illustration of the mechanism underlying hUC-MSCs exosome treatment. Note: DEGs: Differentially expressed genes

By comparing RNA-seq data from injured ovaries of the POI-U groupwith those treated with hUC-MSC exosome, we generated volcano maps of genes that were upregulated and downregulated post-treatment. There were 151 downregulated differentially expressed genes (DEGs) and 49 upregulated DEGs **(**Fig. 7A**)**. GO analysis revealed that the DEGs were enriched in immune and metabolic pathways **(**Fig. 7B**)**. Immune-related genes such as *Cd5, Ccr1, Cd247* and *Tbx21* were highly expressed in the POI-e group, and metabolism-related genes such as *Gal1, Lcmt2, Aass*, *Car2*, and *Aldh1a2* were upregulated in the POI-e group **(**Fig. 7C**)**. Hence, hUC-MSC exosomes may regulate immune and metabolic processes to improve hormonal disorders, oestrous cycles, ovulation disorders and fertility **(**Fig. 7D**)**. *Cao et al*. [22] demonstrated that Adipose mesenchymal stem cell–derived exosome can enhance ovarian function and reproduction of polycystic ovary syndrome via secreting cytokines in metabolism and immunity. However, its specific mechanism needs to be further elucidated.

## Discussion

Clinically, POI is closely related to genes, immune diseases, drugs, surgery, and psychological factors [23, 24]. Long-term HRT is a common treatment strategy [25]. However, clinical data show that long-term HRT can increase the risk of breast cancer [26] and cardiovascular risk [27]. In addition, HRT can only improve patients' endometrial environment and menstrual cycle but cannot improve AMH levels and fertility [25]. Therefore, a reliable therapy to replace HRT treatment is urgently needed.

Suitable and ideal animal models are essential carriers for drug development and mechanism research. Our previous review summarized that an ideal animal model would have the following characteristics: (1) the pathogenic pathways and processes like those observed in humans; (2) the pathological changes in the model can be reversed by drugs; (3) the reproducibility of the results [5]. To mimic the manifestations of POI, we make full use of four animal models (POI-C, POI-B, POI-U, and MS). We demonstrated that POI-C, POI-B, POI-U can effectively mimic the manifestations of POI. Notably, the success rate of POI-U is increased compared to POI-C and POI-B model. The possible reason is that local chemotherapy injections are more toxic to the ovaries compared to systemic injections of chemotherapy drugs. And complications were also dramatically reduced in POI-U group. That's because local intra-ovarian injections of chemotherapy drugs can greatly reduce their retention levels in other organs. Moreover, POI-U requires only a single administration and has fewer adverse reactions to other organs. The strong tolerance of the body and high success rate of POI-U modelling can effectively avoid the multiple organ damage, high death rate, long cycle, complicated operation, and low efficiency caused by systemic administration of chemotherapy drugs. This method is very suitable for the study of chemotherapy drugs to construct a POI animal model. In the future, ultrasound-guided drug injection technology will be more conducive to model construction and drug therapeutic effect studies. Therefore, we further explore the optimal drug therapy in POI-U animal models.

MSCs, especially hUC-MSCs, have been shown to improve hormone levels and fertility in animals with POI in numerous animal experiments since 2012 [28]. Compared to other MSC resources, hUC-MSCs from discarded umbilical cords showed enormous advantage owing to the fewer ethical concerns, painless access, and lack of immunity [29]. A study from 2021, hUC-MSCs can restore the structure and function of damaged ovarian tissue in chemotherapy-induced POI mice and improve fertility. Moreover, the recovery effect of multiple hUC-MSCs transplantation on ovarian function is better than that of single hUC-MSCs transplantation [30]. According to subsequent in-depth research evidence, stem cells improve ovarian function due to their paracrine action (including exosome, cytokines, and growth factors, signalling lipids) rather than differentiation into specific cells.

Compared to hUC-MSCs, exosomes are small and easier to preserve, penetrate the body's thick tissue barrier. Moreover, exosomes can protect their contents from degradation [29]. Exosomes are membranous vesicles secreted by cells via the paracrine pathway and are approximately 40–160 nm in diameter [31]. Accumulating experiments have proven that MSC exosomes play effectively therapeutic roles in Alzheimer's disease [32], bone defect repair [33], polycystic ovary syndrome [34], etc. hUC-MSC exosomes have the functions of inflammatory inhibition, immune regulation, and tissue repair. More importantly, they have the advantages of higher safety, lower immunogenicity and no tumorgenicity [35]. *Kang* et al. demonstrated that hUC-MSC exosomes can alleviate liver injury in non-alcohol related steatohepatitis both in vivo and vitro [35]. hUC-MSC exosomes can regulate the cell–cell communication, cell signalling, and cell or tissue metabolism [29]. *Feng* et al. reported that hUC-MSC exosomes can promote angiogenesis after cerebral ischaemia‒reperfusion injury [36]. However, it is unclear whether hUC-MSC exosomes have beneficial therapeutic effects on ovarian injury and fertility in POI.

The ovary is the most important reproductive organ in women. The ovarian physiological function and reproductive capacity are adversely affected by follicle dysfunction or depletion in POI. In our investigation, hUC-MSC exosomes were successfully extracted and identified. Then, they were injected into POI animal under ultrasound guidance. In *vivo,* we found that hUC-MSC exosomes can effectively enhanced hormone levels, the oestrous cycle, ovarian function. The therapeutic effect of hUC-MSC exosomes on POI rats was mainly due to the improvement in local microenvironment within ovarian tissue, including cellular vitality, inflammation, immune regulation, fibrosis, and metabolism [37]. Addressing fertility issues are the central goal of POI treatment. Our results showed that the reproductive function of POI rat from hUC-MSC exosomes therapy was significantly improved, which collectively suggested that hUC-MSC exosomes showed therapeutic effects on POI rats. However, the long-term effects of hUC-MSC exosomes need to be evaluated, especially the progeny pregnancy rate. At the same time, it is necessary to expand the number of animals in further studies, and more complete results need to be confirmed in preclinical or clinical trials. Elucidating the mechanism of hUC-MSC exosomes-mediated therapy can ensure effective and targeted application of exosomes. Hence, further sequencing revealed that hUC-MSC exosomes might alleviate the symptoms of POI by regulating the ovarian immune and metabolic environment. Of course, more preclinical studies are needed to evaluate its efficacy and safety of hUC-MSC exosomes. Hence, hUC-MSC exosomes may be a potential therapeutic agent for patients with POI.

Importantly, the isolation and purification of hUC-MSC exosomes are complex, and the amounts extracted are limited, posing it a major challenge for clinical applications to achieve adequate amounts and quality control of hUC-MSCs [38]. Second, most hUC-MSC exosomes are administered intravenously to reach the target organ via the homing effect of hUC-MSC exosomes [39, 40]. However, only a small amount of hUC-MSC exosomes reach the treatment site owing to systemic circulation and metabolic function, thus achieving limited therapeutic effects. Therefore, selection of a reasonable administration mode is a major challenge. Ultrasound has the advantages of being visual and convenient [41]. At present, ultrasound-guided in situ injection is increasingly favoured in animal models and preclinical studies [42, 43]. In the ovary, local ovarian injection of methotrexate ultrasound guided via transvaginal injection was applied to the therapy of nontubal ectopic pregnancies [44]. In our study, we innovatively found that local ovarian injection of hUC-MSC exosomes via abdominal ultrasound guidance can effectively treat animals with POI and improve ovarian function.

## Conclusion

In this study, we established POI-C, POI-B, POI-U and MS rat models, among which POI-C, POI-B, POI-U models closely resembling the manifestations of POI. By comprehensively comparing the body weight, ovarian index, status, RGS, complications and model success rate of rat model, we found that POI-U was the optimal animal model, which had high success rate and low complications and mortality. Utilizing the POI-U animal model, we successfully extracted and identified hUC-MSC exosomes and confirmed that ultrasound-guided hUC-MSC exosomes injection can effectively improve the oestrus cycle, hormone levels, pregnancy outcome and ovarian apoptosis in rats with POI. Our study will become fundamental for further clinical conduction of hUC-MSC exosomes-medicated treatment for POI. In addition, ultrasound-guided ovarian local injection of drugs plays a pivotal role in the construction of animal models and hUC-MSC exosome injection. The technique is simple to operate, can reduce the reaction of drugs to the whole body, and achieve certain effects in a single time, which is worthy of further promotion in animal studies. In conclusion, our data propose a novel strategy based on hUC-MSC exosomes may be applied to the treatment of POI disease in the future.

### Supplementary Information

**Additional file 1**. The procedure of cardiac extraction and serum hormone detection. **Fig 1.** The procedure of taking blood from the heart. **Fig 2.** Comparison of serum AMH, FSH, LH, E2, FSH, FSH/LH, T, GnRH, DA and PRL in Con, POI -C, POI-B, POIU and POI-MS groups. **Fig 3.** The comparison of serum AMH, FSH, LH, E2, FSH, FSH/LH, T, GnRH, DA and PRL in POI -C, POI-e and POI -2e group compared with control group.

**Additional file 2**. Heart extraction video.

**Additional file 3**. The video of ultrasound guided in situ ovarian puncture drug injection.

## Data Availability

RNA-seq datasets were generated and updated in the NCBI-GSA (https://ngdc.cncb.ac.cn/gsa/ NCB), GSA number: CRA013502. Additional data are available from the corresponding author on reasonable request.

## Abbreviations

POI: Premature ovarian insufficiency
hUC-MSCs: Human umbilical cord mesenchymal stem cells
POI-C: POI-cyclophosphamide
POI-B: POI-cyclophosphamide + busulfan
POI-U: POI-ultrasonic guidance cyclophosphamide injection
MS: Maternal separation
POI-e: Ultrasound-guided hUC-MSCs exosome injection
AMH: Anti-müllerian hormone
LH: Luteinizing hormone
FSH: Follicle-stimulating hormone
DA: Dopamine
T: Testosterone
PRL: Prolactin
GnRH: Gonadotropin-releasing hormone
DEGs: Differentially expressed genes
GO: Gene Ontology
