# Supplementary material for: Model construction and drug therapy of primary ovarian insufficiency by ultrasound-guided injection
Source: Stem Cell Res Ther. 2024 Feb 20;15:49. doi: 10.1186/s13287-024-03646-y (PMC10880334; doi:10.1186/s13287-024-03646-y)
Supplement: Supplementary file 1 — Additional file 1. The procedure of cardiac extraction and serum hormone detection. Fig 1. The procedure of taking blood from the heart. Fig 2. Comparison of serum AMH, FSH, LH, E2, FSH, FSH/LH, T, GnRH, DA and PRL in Con, POI -C, POI-B, POIU and POI-MS groups. Fig 3. The comparison of serum AMH, FSH, LH, E2, FSH, FSH/LH, T, GnRH, DA and PRL in POI -C, POI-e and POI -2e group compared with control group. [file 13287_2024_3646_MOESM1_ESM.docx]

**Supplementary figures and video**

**Supplementary video 1:** Heart extraction video

**Supplementary video 2:** Ultrasound guided in situ ovarian puncture drug injection

**RNA-seq data:** https://www.omicsmart.com/RNAseq/home.html#/group/list


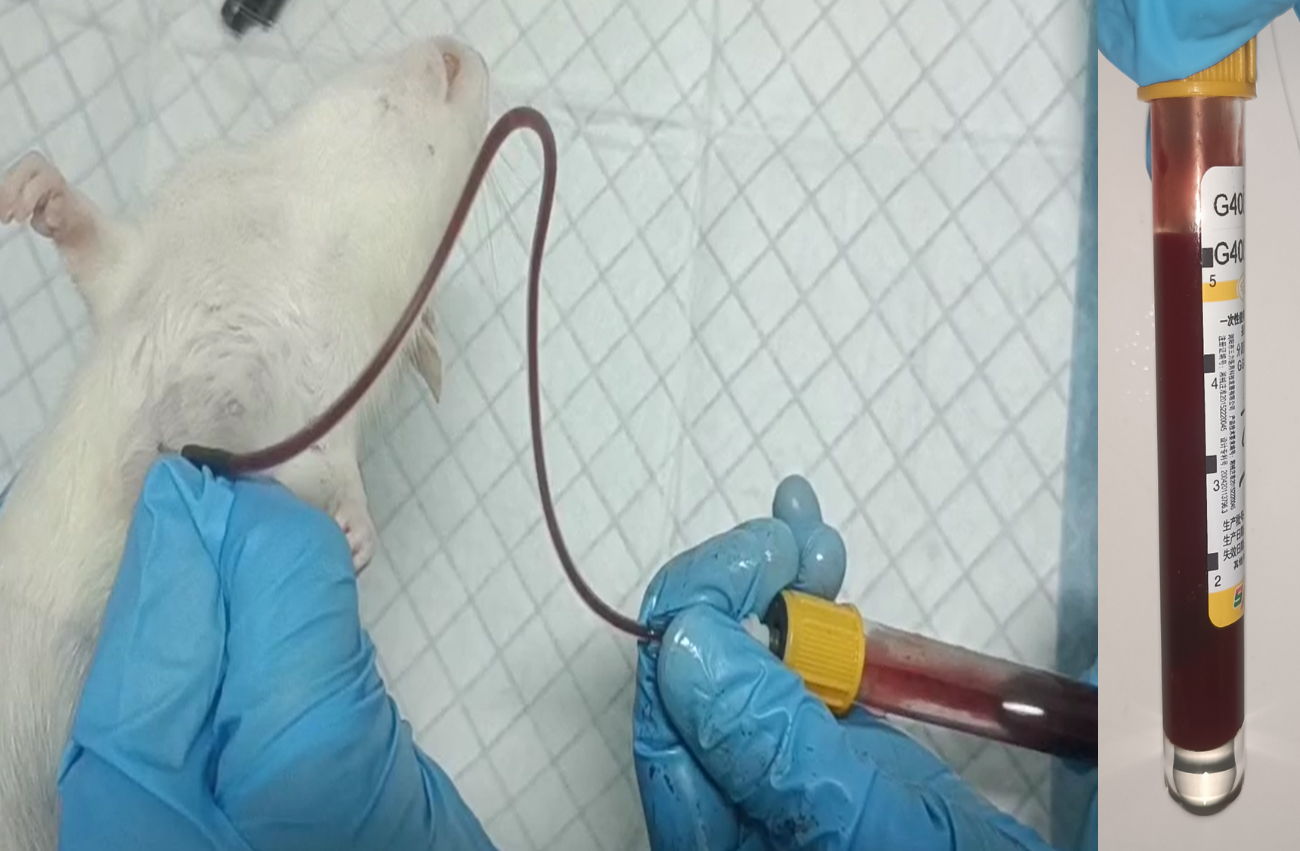


**Supplementary figure 1. The procedure of taking blood from the heart**


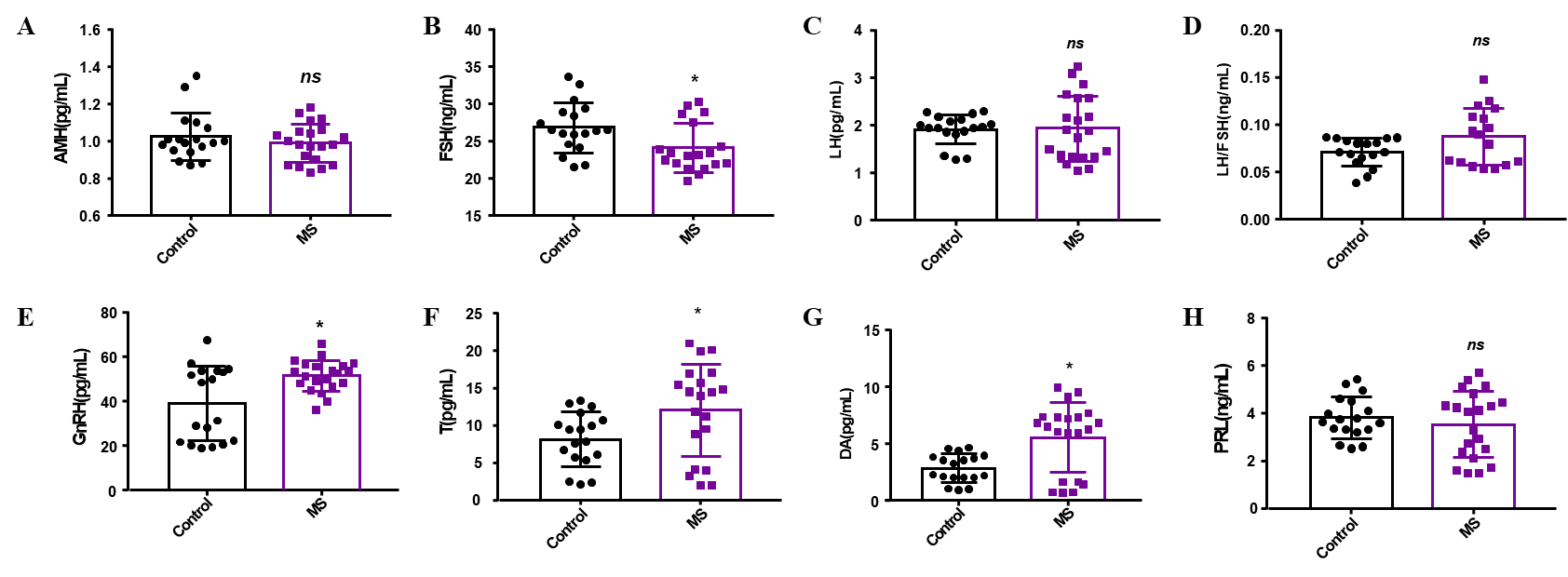


**Supplementary figure 2.** Comparison of serum AMH, FSH, LH, E2, FSH, FSH/LH, T, GnRH, DA and PRL in Con, POI -C, POI-B, POI-U and POI-MS groups.


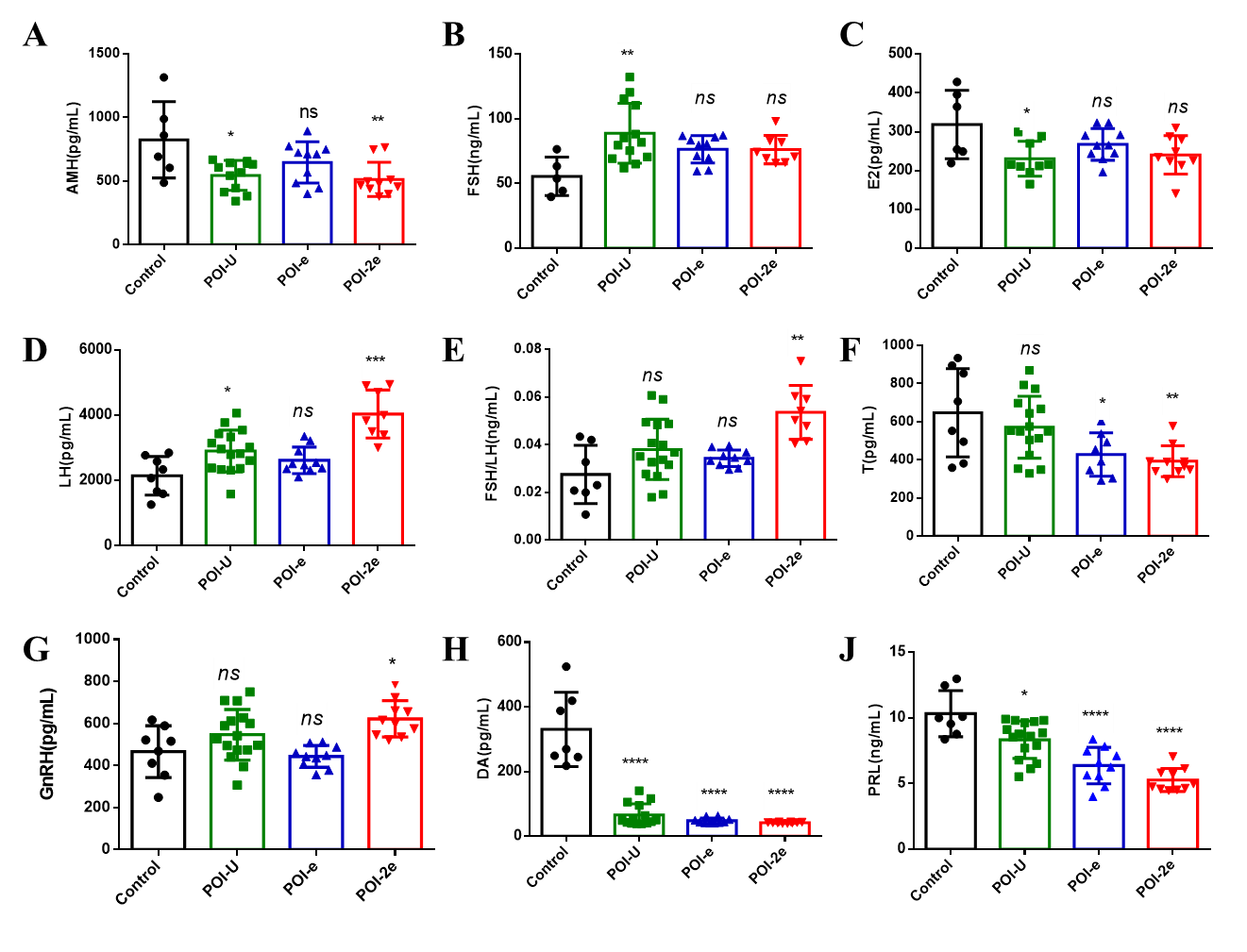


**Supplementary figure 3**. Comparison of serum AMH, FSH, LH, E2, FSH, FSH/LH, T, GnRH, DA and PRL in groups Con, POI -C, POI-e and POI -2e.
